# Metabolomics analysis reveals serum biomarkers in patients with diabetic sarcopenia

**DOI:** 10.3389/fendo.2023.1119782

**Published:** 2023-03-22

**Authors:** Yuwei Tan, Xiaosong Liu, Yinping Yang, Baoying Li, Fei Yu, Wenqian Zhao, Chunli Fu, Xin Yu, Zhenxia Han, Mei Cheng

**Affiliations:** ^1^ Department of Geriatric Medicine, Qilu Hospital, Cheeloo College of Medicine, Shandong University, Jinan, China; ^2^ Key Laboratory of Cardiovascular Proteomics of Shandong Province, Qilu Hospital, Cheeloo College of Medicine, Shandong University, Jinan, China; ^3^ Jinan Clinical Research Center for Geriatric Medicine (202132001), Jinan, China; ^4^ Jinan Aixinzhuoer Medical Laboratory, Jinan, China

**Keywords:** diabetes, sarcopenia, metabolomics, biomarkers, serum metabolites

## Abstract

**Introduction:**

Diabetic sarcopenia (DS) is characterized by muscle atrophy, slower nerve conduction, reduced maximum tension generated by skeletal muscle contraction, and slower contraction rate. Hence, DS can cause limb movement degeneration, slow movement, reduced balance, reduced metabolic rate, falls, fractures, etc. Moreover, the relevant early biological metabolites and their pathophysiological mechanism have yet to be characterized.

**Method:**

The current cross-sectional study employed serum metabolomics analysis to screen potential noninvasive biomarkers in patients with diabetic sarcopenia. A total of 280 diabetic patients were enrolled in the study (n = 39 sarcopenia [DS], n = 241 without sarcopenia [DM]). Ten patients were randomly selected from both groups. Non-targeted metabolomic analysis was performed by ultra-high-performance liquid chromatography-electrospray ionization tandem mass spectrometry.

**Results:**

A total of 632 differential metabolites were identified, including 82 that were significantly differentially abundant (*P* < 0.05, VIP > 1, FC > 1.2 or FC < 0.8). Compared with the DM group, the contents of pentadecanoic acid, 5'-methylthioadenosine (5'-MTA), N,N-dimethylarginine (asymmetric dimethylarginine, ADMA), and glutamine in the DS group were significantly increased, while that of isoxanthohumol was decreased.

**Discussion:**

Based on receiver operating characteristic curve analysis, pentadecanoic acid, 5'-MTA, ADMA, and glutamine may serve as potential biomarkers of DS. Moreover, ATP-binding cassette (ABC) transporters and the mammalian target of the rapamycin signaling pathway were found to potentially have important regulatory roles in the occurrence and development of DS (P < 0.05). Collectively, the differential metabolites identified in this study provide new insights into the underlying pathophysiology of DS and serve as a basis for therapeutic interventions.

## 1 Introduction

Type 2 diabetes mellitus (T2DM) is a chronic multifactor metabolic disease. The associated chronic hyperglycemia and insulin resistance can induce downstream metabolic changes, resulting in the imbalance of protein, lipid and carbohydrate metabolism, resulting in cardiovascular and cerebrovascular diseases (1), neurodegenerative diseases (2), muscle atrophy (3) and other systemic multi-organ chronic damage and even failure. Consequent detrimental effects occur in muscle mass and muscle strength, thus, significantly impacting the musculoskeletal system and leading to the development of sarcopenia (4, 5). Indeed, the prevalence of sarcopenia is high in patients with T2DM, ranging from 7% to 29.3% (6). However, the pathogenesis and etiology of diabetic sarcopenia (DS) are complex (7), with nutritional deficiency (8), muscle fat accumulation (9, 10), inflammation (11), oxidative stress (9, 12), lack of exercise (13), and diabetic complications such as diabetic microangiopathy (14), diabetic neuropathy (15), and diabetic foot disease (16) reported as potential etiologic factors. DS is characterized by muscle atrophy, slower nerve conduction, reduced maximum tension generated by skeletal muscle contraction, and gradually slower contraction rates. Moreover, with an increase in age, middle-aged and elderly individuals with DS further experience limb movement degeneration, reduced balance, and metabolic rate, as well as increased risk of falls, fractures, etc. Furthermore, although DS is a complication of T2DM with high incidence and different treatment methods (11), diagnostic criteria have only been established for sarcopenia, while that for DS remains unclear.

Metabolomics analysis effectively detects and quantifies small molecules, including metabolites, within samples to identify differentially abundant metabolites across various disease states or physiological periods (17). In particular, it can be applied to detect early markers of disease, discover drug targets, elucidate disease mechanisms, and facilitate improved disease diagnosis. Currently, the diagnostic criteria for DS primarily focus on assessing skeletal muscle strength, mass, etc., with no established serological indicators to guide diagnosis. In this study, metabolomic analysis is employed to screen serum metabolomic markers of DS and elucidate potential underlying mechanisms associated with DS development and pathogenesis. Hence, the primary aim of this study is to identify targets for early clinical diagnosis and treatment of DS, as well as potential markers capable of predicting disease prognosis and development.

## 2 Materials and methods

### 2.1 Study population

A total of 280 patients aged > 50 years were consecutively recruited from the Department of Geriatric Medicine, Qilu Hospital, Shandong University, Shandong Province, China, comprising diabetic patients with sarcopenia (DS, *n* =39) and without sarcopenia (DM, *n* = 241) were enrolled. From this patient population, we randomly selected ten patients from the DS and ten from the DM groups. T2DM was defined based on the standard criteria set by the American Diabetes Association of the Endocrinology Department (18). Sarcopenia was defined, based on the criteria recommended by the Asian Working Group for Sarcopenia (AWGS) (19), as low muscle mass with no effect on muscle strength or physical performance. Appendicular skeletal muscle mass (ASM) index (ASM/height2) <  7.0 kg/m^2^ in men and ASMI  <  5.7 kg/m^2^ in women was defined as low muscle mass, and handgrip strength < 26 kg in men and < 18 kg in women was defined as low muscle strength. Low physical performance was defined as a gait speed < 0.8 m/s. Exclusion criteria included a history of stroke, stents, artificial pacemaker, serious bone, joint, or neuromuscular diseases, mental diseases, metal implants, or a history of malignant tumors, liver disease, end-stage kidney disease, thyroid dysfunction, arthritis, carpal tunnel syndrome, serious diabetic complications (kidney failure, diabetic retinopathy, diabetic foot, etc.), bariatric surgery, or lower limb surgery. Trained interviewers collected all questionnaire data.

This study was approved by the Medical Ethics Committee of the Qilu Hospital of Shandong University (Approval No: 2021274). All methods and analyses were carried out according to approved protocols and guidelines. All study participants provided written informed consent.

A general questionnaire was used to collect data related to age, sex, and medical history. Standardized equipment was used to measure patient height and weight. Participants wore light clothing and removed their shoes during measurements. Body mass index (BMI) was calculated by dividing the weight (kg) by height squared (m)^2^. The patient was placed in the supine position with an appropriate pressure cuff placed above the anterior cubital fossa to measure systolic blood pressure (BP) and diastolic BP in both arms; the lower right and left systolic BP and diastolic BP were measured using a sphygmomanometer in the supine position. Systolic BP and diastolic BP was measured at the levels of the posterior tibial and tibial arteries. The ankle brachial index (ABI) for both lower limbs was calculated.

### 2.2 Measurement of skeletal muscle mass, muscle strength, and physical performance

The regional body composition of patients with diabetes was determined using INBODY720. Bioimpedance analysis (BIA) was employed to screen whole-body muscle mass. Body composition was analyzed using a multi-frequency bioelectrical impedance analysis device (InBody (720) body composition analyzer, Biospace Co., Ltd., Seoul, South Korea); participants fasted the night prior and sat quietly within the device for 5 min before measurements were made. Segmental resistance was measured at six frequencies (1, 5, 50, 250, and 500 Hz, and 1 MHz); output was recorded. The ASMI was calculated by dividing the ASM by height squared in meters.

Handgrip strength was used to assess muscle strength. Grip strength was measured using The Jamar dynamometer (Sammons Preston, Inc., Bolingbrook, IL, USA) in the standard postures recommended by the American Association of Hand Therapists (ASHT)(20). Grip strength measurements were recorded for both arms of each patient and repeated thrice until the maximum load was reached.

### 2.3 Metabolomic analysis

#### 2.3.1 Sample collection

Fasting blood samples were collected between 7:00 and 8:00 a.m. from the antecubital vein and stored in a Progel tube in a cryogenic rack (2°C–8°C). The whole blood was immediately centrifuged at 1000 × g for 10 min at room temperature to separate the serum. Serum samples were immediately stored at -80°C for metabolomic analysis.

#### 2.3.2 Metabolomic analyses

After thawing at 4°C, 100 μL of each sample was extracted. The mixture was prepared using 800 µL of pre-cooled methanol/acetonitrile (1:1, v/v) and 100 µL pre-cooled water, subjected to 1 h ultrasonic treatment in an ice bath, and then incubated with the sample at -20 °C for 2 h. After centrifugation at 4°C and 16,000 *× g* for 20 min, the supernatant was collected and drained using a high-speed vacuum centrifuge. When mass spectrometry was required, the sample was redissolved with 100 µL of methanol-aqueous solution (1:1, v/v), and the supernatant was collected for sample analysis following centrifugation at 20,000 *× g* and 4°C for 20 min.

Non-targeted metabolomics analysis was performed using the DS. SHIMADZU-LC30 UHPLC system with an ACQUITY UPLC^®^ HSS T3 column (2.1 × 100 mm, 1.8 µm; Waters, Milford, MA, USA). The samples were separated by UPLC and analyzed by mass spectrometry using a QE Plus mass spectrometer (Thermo Scientific). The HESI source was employed for ionization. Metabolite structure was identified by accurate mass number matching (mass tolerance < 20 ppm) and secondary spectrogram matching (mass tolerance < 0.02 Da). The HMDB, MassBank, and other public databases, as well as the local metabolite standard database, were searched, and the MSDIAL software (RIkagaku KENkyusho/Institute of Physical and Chemical Research, Japan) was employed to conduct retention time correction, peak alignment, and peak area extraction. For the extracted data, the missing values in the group were deleted; 50% ion peaks were not included in subsequent statistical analysis. The total peak area of the positive and negative ion data was normalized, and the peaks were integrated; R software was used for pattern recognition. After the data were subjected to unit variance scaling (UV), subsequent data analysis was conducted.

#### 2.3.3 KEGG enrichment analysis

The Kyoto Encyclopedia of Genes and Genomes (KEGG; www.genome.jp/kegg/pathway.html) database was used to analyze the metabolite data, and Fisher’s exact test was used for enrichment analysis. After performing false discovery rate (FDR) correction for multiple comparisons, the significantly enriched pathways (*P* < 0.05) were screened.

#### 2.3.4 Differential metabolite identification

All multivariate data analysis and modeling used R (version: 4.0.3) and R packages. The data was centered on the mean, and Pareto scaling was adopted. Principal component analysis (PCA) and orthogonal partial least squares discriminant analysis (OPLS-DA) were used to establish the model. All evaluation models were overfitted *via* permutation test. At the univariate analysis level, the metabolites were identified based on the statistical significance threshold for the variable influence of the projected (VIP) values obtained in the OPLS-DA model and by a two-tailed Student’s *t*-test (*P*-value) for normalized raw data. In multigroup analysis, one-way analysis of variance (ANOVA) was used to calculate *P* values. Metabolites with VIP values > 1.0, FC > 1.2 or < 0.8, and *P* values < 0.05 were considered statistically significant. All identified differential metabolites were applied for cluster analysis using R package.

### 2.4 Laboratory measurements

Fasting blood samples were collected and centrifuged to separate serum (12,000 *× g*, 10 min, 4°C). Glucose(Glu), triglyceride (TG), total cholesterol (TC), high-density lipoprotein (HDL) cholesterol, low-density lipoprotein (LDL) cholesterol (chemiluminescent immunoassay, Roche), and glycosylated hemoglobin (HbA1c; high-performance liquid chromatography assay, HPLC-873G8, Tosoh) were measured by the laboratory of Qilu Hospital of Shandong University.

### 2.5 ELISA detection

A total of 280 serum samples were collected according to section 2.3.1 (39 from the DS group and 241 from the DM group) for ELISA using a Human 5′-Methylthioadenosine (MTA) ELISA Kit (MM-61971H, FEIYA Biotechnology, Jiangsu, China) and Human Asymmetric Dimethylarginine (ADMA) ELISA Kit (MM-51745H1, FEIYA Biotechnology). The assay was carried out according to the manufacturer’s instructions. The absorbance (OD value) was measured at a wavelength of 450 nm with a microplate reader, and the concentration of the target substance was calculated using a standard curve.

### 2.6 Statistical analysis

In this study, SPSS 23.0 software was used for statistical analysis, and the S-W method was used to assess data normality. Measurement data conforming to normal distribution were expressed in the form of “mean ± standard deviation”, and an independent sample t-test was used to analyze differences between the two groups. Count data were expressed as a “composition ratio,” and the Chi-square test was used to analyze the difference between the two groups. All *P* < 0.05 were considered statistically significant.

## 3 Results

### 3.1 Characteristics of study participants

The clinical characteristics of diabetic patients with DS are presented in **Table 1**. No significant differences were observed in sex, however, significant differences were detected in muscle mass (*P* < 0.05), grip strength (*P* < 0.01), and calf/waist/hip circumference (*P* < 0.05) between the DS and DM groups. Moreover, compared with the DM control group, DS patients were older and had a longer course of T2DM (*P* < 0.05). DS patients also had lower BMI and DBP, as well as higher pulse pressure (*P* < 0.05), serum triglyceride concentrations, plasma glucose, and glycosylated hemoglobin levels (*P* < 0.05 for each). Moreover, serum HDL levels were lower in DS patients compared with those in the DM controls (*P* < 0.05). Meanwhile, no significant differences were observed in uric acid concentration.

**Table 1:** Clinical characteristics of participants in the diabetic patients with sarcopenia (DS) and diabetic patients without sarcopenia (DM) control groups.

Characteristic	DM (*n* = 10)	DS (*n* = 10)	*P*-value^a^
Age, years	69.90 ± 6.56	78.30 ± 8.65	0.033
Female/male	7/3	4/6	0.178
Body Mass Index (BMI), kg/m^2^	21.76 ± 1.48	19.93 ± 1.62	0.022
Course	16.20 ± 2.64	22.20 ± 4.26	0.003
Systolic Blood Pressure	126.30 ± 17.41	136.20 ± 19.57	0.272
Diastolic Blood Pressure	74.60 ± 10.62	64.40 ± 9.35	0.045
pulse pressure	51.70 ± 14.79	71.80 ± 16.42	0.014
Glucose, mmol/L	6.81 ± 1.49	8.23 ± 1.25	0.043
Triglycerides, mg/dL	1.25 ± 0.32	1.62 ± 0.29	0.018
Total cholesterol, mg/dL	4.26 ± 0.68	4.60 ± 0.61	0.275
High-density lipoprotein cholesterol, mg/dL	1.35 ± 0.30	1.04 ± 0.14	0.013
Low-density lipoprotein cholesterol, mg/dL	2.36 ± 0.64	2.44 ± 0.75	0.808
Uric Acid	302.90 ± 48.73	310.30 ± 48.90	0.751
Glycosylated hemoglobin, %	6.71 ± 1.08	7.86 ± 1.12	0.040
Ankle Brachial Index	1.12 ± 0.10	1.00 ± 0.10	0.027
Waist circumference, cm	93.10 ± 5.52	86.90 ± 6.50	0.043
Hip circumference, cm	102.90 ± 5.39	97.50 ± 4.84	0.038
Waist-to-hip ratio	0.90 ± 0.03	0.89 ± 0.05	0.509
Left calf circumference, cm	34.90 ± 1.45	28.50 ± 1.75	0.000
Right calf circumference, cm	35.10 ± 1.81	29.00 ± 2.24	0.000
Grip strength, kg	24.76 ± 2.58	12.73 ± 2.37	0.000
Appendicular skeletal muscle mass [ASM], kg	25.90 ± 4.44	19.54 ± 3.92	0.005
ASM index, kg/height^2^	7.98 ± 0.85	5.72 ± 0.94	0.000
Appendicular lean mass to BMI ratio	1.19 ± 0.17	0.99 ± 0.22	0.048
Insulin(%)	50	40	0.653
Biguanides(%)	30	30	1
Sulfonylureas(2^nd^ generation) (%)	30	40	0.639
α-Glucosidase inhibitors(%)	10	10	1
Others^b^(%)	20	30	0.605

a P-values were analyzed using t-test. Values are presented as mean (± SD) or number (percentage).

b Others: DPP‐IV inhibitor, dipeptidyl peptidase - IV inhibitor; GLP-1 RA, glucagon-like peptide 1 receptor agonist; SGLT2, sodium-glucose cotransporter-2; Thiazolidinedione.

ASM, appendicular skeletal muscle mass; BMI, body mass index.

### 3.2 Serum metabolic profiling using ultra high-performance liquid chromatography coupled with quadrupole-exactive mass spectrometry

**Figure 1:**
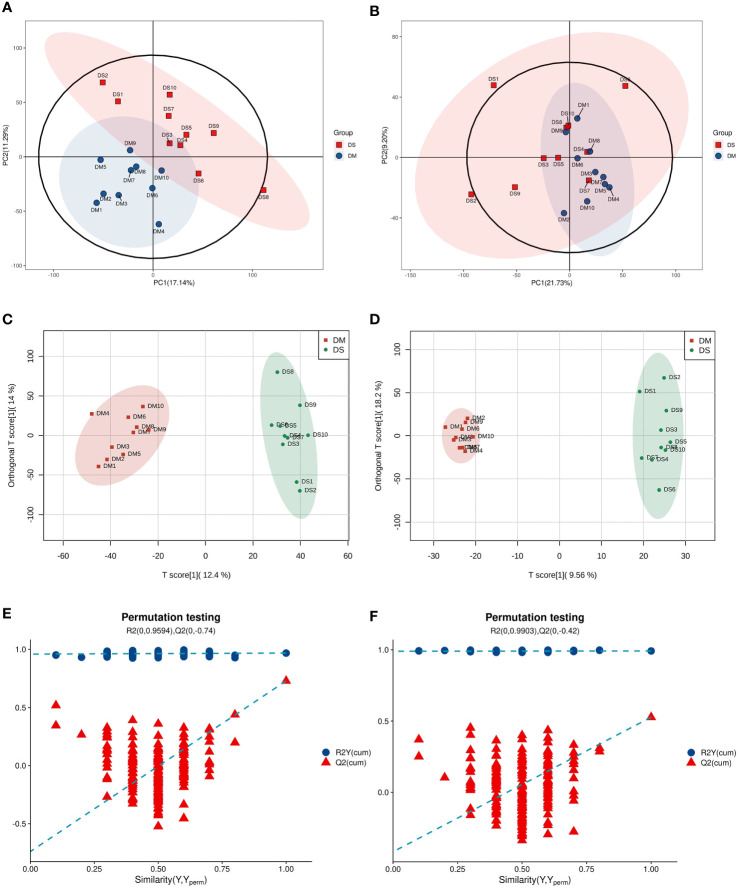
Multivariate statistical analysis **(A)** Principal component analysis (PCA) scatter plot of DS (green circle) *vs.* DM (red circle) in positive ion mode. **(B)** PCA scatter plot of DS (green circle) *vs.* DM (red circle) in the negative ion mode. **(C)** Orthogonal partial least squares discriminant analysis (OPLS-DA) in positive ion mode of DS *vs.* DM. **(D)** OPLS–DA in negative ion mode of DS *vs*. DM. **(E)** OPLS–DA permutation test in positive ion mode. **(F)** OPLS–DA permutation test in negative ion mode.

The overall distribution trend of all samples was observed through PCA analysis (**Figures 1A, B**). The total variance of the data represented by the first two principal components in positive ion mode was 17.14% (**Figures 1A**). In negative ion mode, the first two principal components represented 21.73% of the total variance in the data (**Figure 1B**). The PCA score of the positive-ion group showed a significant separation tendency, whereas an insignificant trend was observed in the separation of PCA scores between negative ion groups. Considering the blank supervision of the PCA model, OPLS-DA was subsequently performed to further filter the differential metabolites between groups (**Figures 1C, D**). The OPLS-DA model (R2Y, Q2) in positive ion mode (R2Y = 0.968, Q2 = 0.729) and negative ion mode (R2Y = 0.992, Q2 = 0.527) revealed that the R2Y and Q2 were ≥ 0.5, indicating that the model was stable and reliable with good predictive capacity. The permutation test of the OPLS-DA model established in positive and negative ion modes is shown in **Figures 1E, F**. OPLS-DA model overfitting did not occur and exhibited significant differences between sample distributions, indicating the presence of significant differences in serum metabolites between the two groups. From all PCA and OPLS-DA scores, it can be speculated that the occurrence of DS correlates with changes in the levels of certain metabolites.

**Figure 2:**
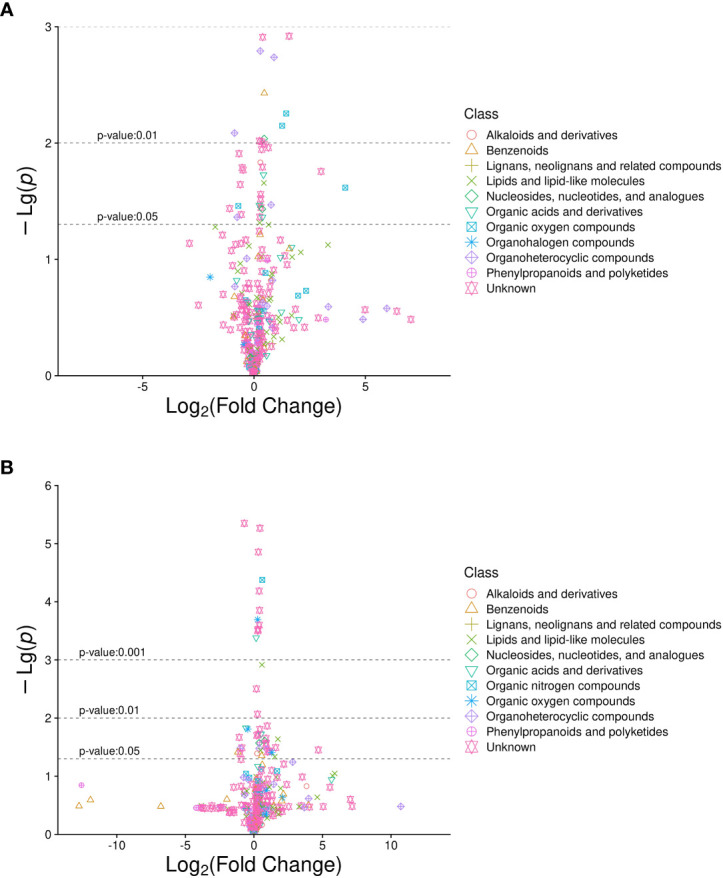
Volcano plot of differentially abundant metabolites in diabetic patients with sarcopenia (DS) DS *vs.* diabetic patients without sarcopenia (DM). **(A)** DS *vs.* DM in positive ion mode. **(B)** DS *vs.* DM in negative ion mode. The abscissa is the multiple change value of the expression difference in metabolites between the two groups, and the ordinate is the statistical test value of the expression difference in metabolites, i.e., *P*-value. The abscissa and ordinate values were processed logarithmically. Each point represents a specific metabolite.

**Figure 3:**
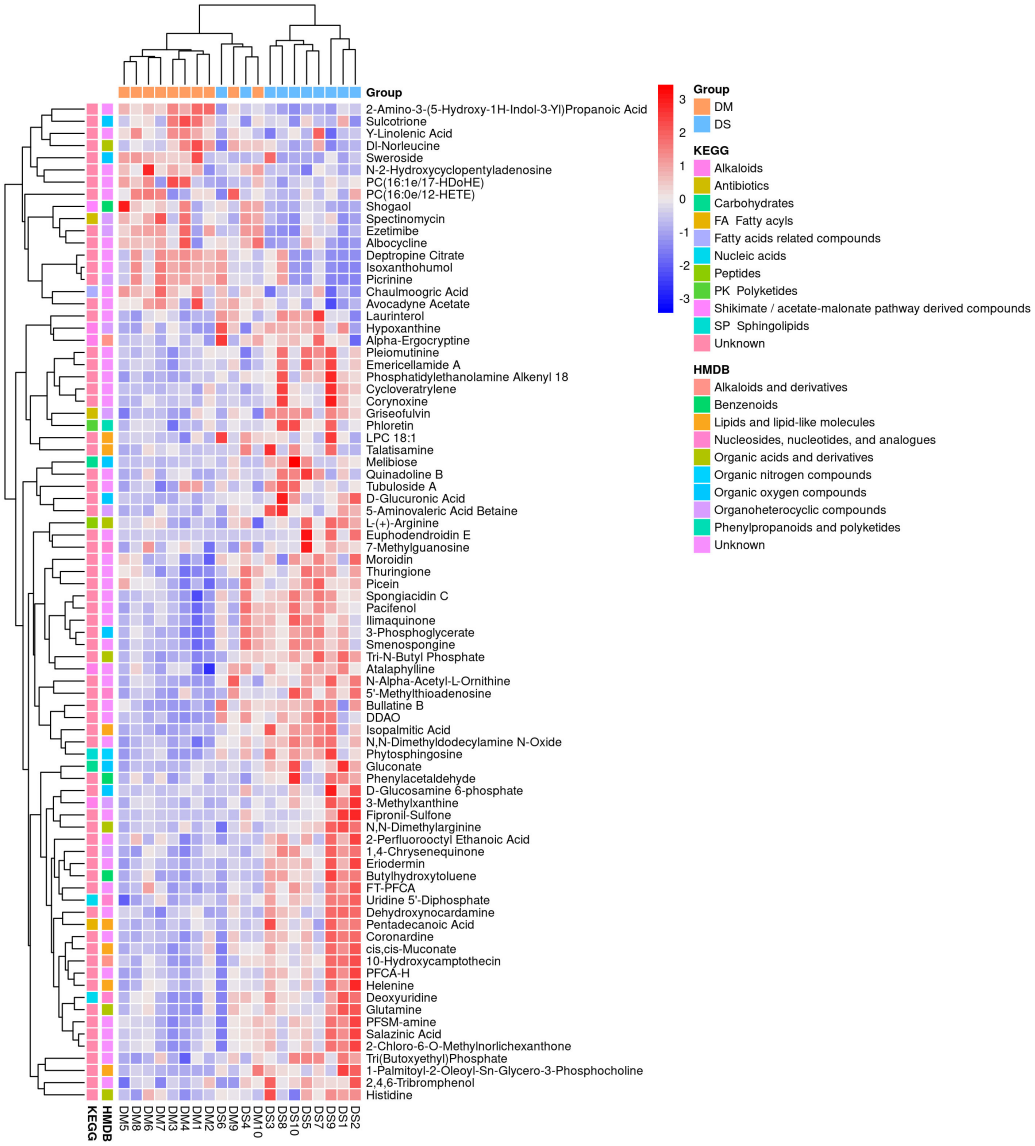
Heat map analysis of 82 differential metabolites between the DS and DM groups. The values of differential metabolites were normalized and shown on a color scale. The high and low metabolite levels are represented as red and blue scales, respectively.

A total of 632 differential metabolites were detected (**Figures 2A, B**), including 82 significant differential metabolites (*P* < 0.05, VIP > 1), primarily classified as alkaloids and their derivatives, benzenoids, lignans, neolignans, and related compounds; lipids and lipid-like molecules; nucleosides, nucleotides, and analogs; organic acids and their derivatives; organic nitrogen compounds; organic oxygen compounds; organoheterocyclic compounds; and phenylpropanoids and polyketides. The predominant differential metabolites included D-glucosamine 6-phosphate, D-glucuronic acid, melibiose, N,N-dimethylarginine (asymmetric dimethylarginine, ADMA), etc. Qualitatively significant differentially abundant metabolites were then applied to construct a hierarchical cluster map for each group of samples (**Figure 3**). The clustering results were then used to assess the stability of the expression of selected target metabolites within each group. Meanwhile, the metabolites within similar clusters were deemed to have identical expression patterns, which might indicate a corresponding reaction step in the metabolic process.

### 3.3 ROC analysis of potential DS diagnostic biomarkers

**Figure 4:**
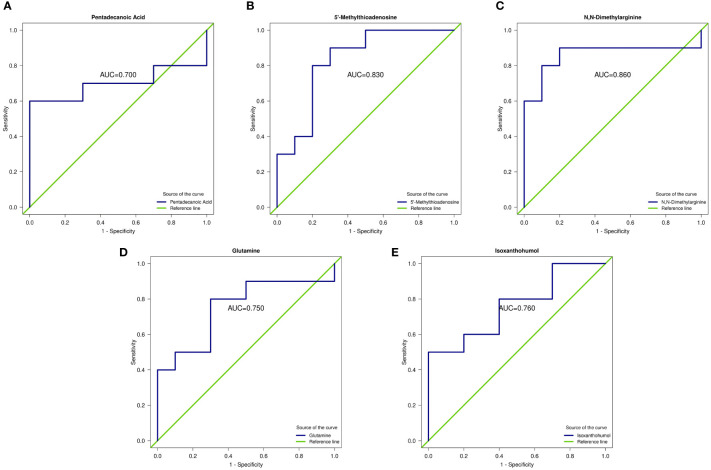
Receiver operating characteristic curve (ROC) of differential metabolites. **(A–E)** ROC curves of five metabolites: pentadecanoic acid, 5’-methylthioadenosine (MTA), N,N-dimethylarginine (asymmetric dimethylarginine, ADMA), glutamine, and isoxanthohumol (IX). AUC represents the recognition performance. The 95% confidence interval of the AUC was calculated based on the nonparametric resampling method. The points on the curve represent the optimal thresholds determined by the ROC curve to distinguish the two groups. The ordinate is the enrichment rate, which is the ratio of the amount of metabolites enriched in the pathway to the amount of metabolites annotated in the pathway.

The area under the ROC curve (AUC) for d-glucuronic acid and pentadecanoic acid was 0.94 and 0.700, respectively, whereas 5’-methylthioadenosine (5’-MTA) was 0.83. The concentration of ADMA was 0.86, glutamine was 0.75, and isoxanthohumol (IX) was 0.76 (*P* < 0.05; **Table 2** and **Figures 4A-E**).

**Table 2:** Predictive capacity of each metabolite for DS.

Metabolite	AUC	*P*-value	FC	Log2FC	Cluster	State
Pentadecanoic acid	0.70	0.022	1.36	0.44	5	up
5’-Methylthioadenosine	0.83	0.024	1.71	0.78	3	up
N,N-Dimethylarginine	0.86	0.019	1.44	0.52	3	up
Glutamine	0.75	0.043	1.30	0.38	3	up
Isoxanthohumol	0.76	0.021	0.47	-1.08	3	down

AUC, area under the receiver operating characteristic curve.

### 3.4 Detection and identification of differential metabolites

**Figure 5:**
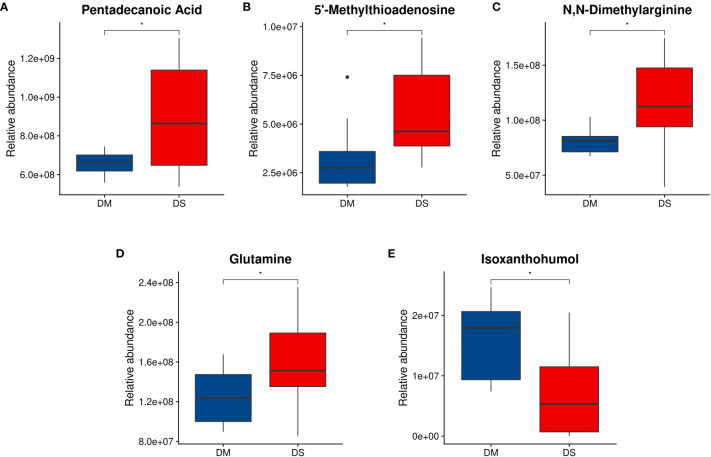
Analysis of differential metabolite abundance. **(A)** Quantitative differences between DS and DM controls for plasma pentadecanoic acid concentrations; **(B)** 5’-methylthioadenosine concentrations; **(C)** N,N-dimethylarginine concentrations; and **(D)** glutamine concentrations. **(E)** Isoxanthohumol concentration. Welch t-test was performed to compare the means of each metabolite in participants with DS and DM controls. * *P* < 0.05 *vs.* control. Compared with the DM group, the concentrations of hexadecanoic acid, methionine, N,N-dimethylarginine, glutamine, and gluconic acid in the DS group increased, and the concentration of isoxanthohumol decreased.

**Figure 6:**
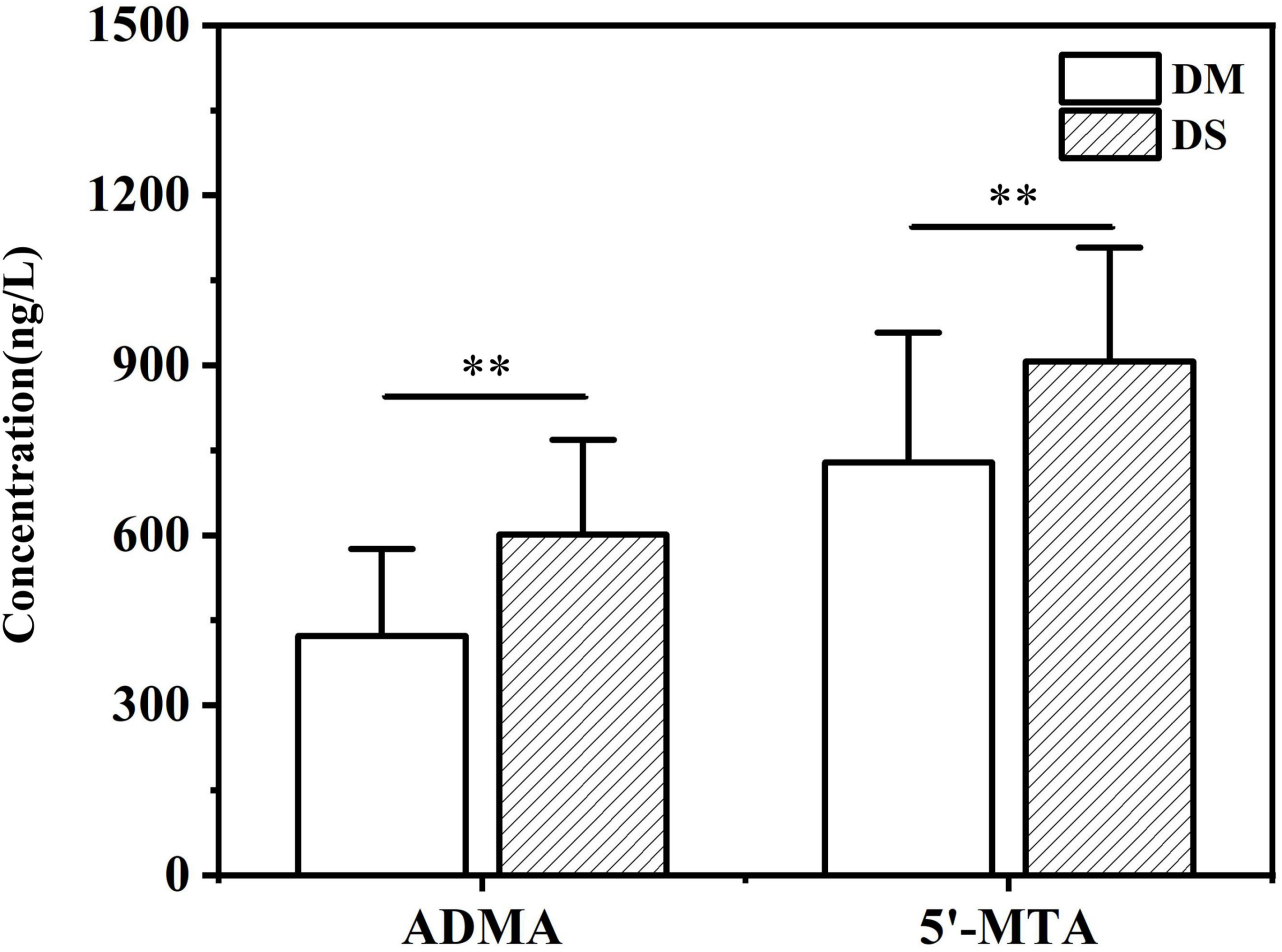
ELISA analysis of the concentration of serum ADMA and 5’-MTA in DS and DM group. ** *P* < 0.01 vs. DM group.

Compared with the DM group, the concentrations of pentadecanoic acid, 5’-MTA, ADMA, glutamine and gluconic acid in the DS group were significantly increased, while that of IX was decreased(*P* < 0.05 for each, **Figures 5A-E**). We further determined the concentration of ADMA and 5’-MTA in the serum of 280 patients using ELISA. ELISA results showed that the concentrations of 5’-MTA and ADMA were significantly increased in the DS group (*P* < 0.01, **Figure 6**), which were consistent with the results of metabolomics analysis.

### 3.5 KEGG pathway enrichment analysis

**Figure 7:**
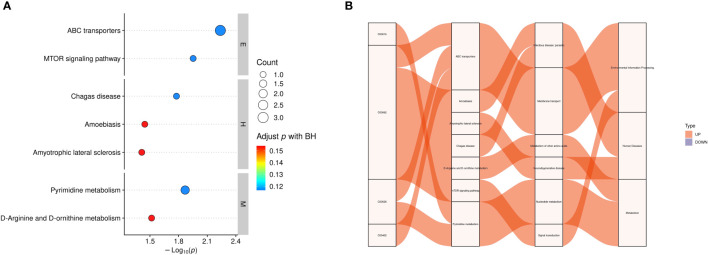
Functional analysis of differential metabolites. **(A)** KEGG enrichment statistics. The x-axis indicates the negative log transformation of the *P*-value, and the y-axis indicates the name of the KEGG metabolic pathway. The size and color of the bubbles represent the number and degree of enrichment of different metabolites, respectively. **(B)** Sankey diagram: the first column on the left represents the differential metabolites that were upregulated or downregulated, and the height of the box represents the amount of data flow, that is, the number of metabolites annotated to pathways. The more corresponding pathways, the higher the box. The red streamline represents the flow direction of upregulated (up) metabolites, whereas the blue streamline represents the flow direction of downregulated (down) metabolites. The second, third, and fourth columns represent the pathways, and the hierarchy increases accordingly. M: metabolism, E: environmental information processing, H: human diseases, C00015: uridine 5’-diphosphate, C00062: L-(+)-arginine, C00526 deoxyuridine, C05402: melibiose; BH: biological process.

We used KEGG enrichment analysis to identify the relevant metabolic pathways involved in DS, and the results showed that the mammalian target of rapamycin (mTOR) signaling pathway, ABC transporters, amyotrophic lateral sclerosis, amoebiasis, pyrimidine metabolism, as well as D-arginine, and D-ornithine metabolism pathways were the primary pathways associated with DS (*P* < 0.05, **Figure 7**). KEGG enrichment analyses were carried out with the Fisher’s exact test, and FDR correction for multiple testing was performed.KEGG analysis further identified ABC transporters (*P* = 0.005789, Corrected = 0.116732)and the mTOR signaling pathway (*P* = 0.011146, Corrected = 0.116732) as the most significantly different between the two groups.

## 4 Discussion

In this study, we randomly selected ten DS and ten DM patients for serum untargeted metabolomics analysis. The concentrations of alkanoic acid, 5 ‘-MTA, ADMA, and glutamine were significantly increased in the DS group compared with the DM group, while that of IX was significantly decreased. KEGG analysis further identified ABC transporters and the mTOR signaling pathway as significantly different between the two groups. To our knowledge, this is the first study to report biomarkers for DS using a metabolomics approach. To identify serum metabolomic markers of DS and explore the related mechanisms, muscle testing was performed on enrolled patients and clinical characteristics were statistically analyzed. The results showed that, compared with the DM group, DS patients were older, had lower BMI, and significantly lower grip strength and muscle mass. Moreover, serum laboratory tests showed significant differences in HbA1c, blood glucose, and blood lipid levels between the two groups, which is consistent with previously reported results (21).

Additionally, higher levels of pentadecanoic acid were detected in the DS group compared with the DM group. As a long-chain saturated fatty acid (LCFA), pentadecanoic acid is a potent biological marker of dairy fat intake (22). As LCFAs accumulate, they become deposited in muscle, consequently compromising muscle integrity. In fact, within muscle tissue, continuous accumulation of LCFAs can lead to disordered protein synthesis and lipotoxicity, activation of immune cells, and induction of muscle cell inflammation, resulting in muscle atrophy and apoptosis of muscle proteins (23). Meanwhile, in non-adipose tissue, ectopic accumulation of LCFAs leads to metabolic dysfunction; increased lipid content causes increased lipid load, muscle atrophy, and a corresponding decrease in muscle content and mass (24). It has been suggested that damage to muscle integrity may be related to intramuscular fat (peroxisome proliferator-activated receptor γ)(25) and intramuscular components associated with the resulting muscle inflammation (cyclo-oxygen-ase-2, inducible nitric oxide synthase) (26). This further suggests a relationship between ectopic intramuscular adipose and muscle loss. In addition, LCFAs can lead to insulin resistance, which is one of the primary etiologic factors of sarcopenia (27). Hence, the LCFA pentadecanoic acid accumulates in skeletal muscle, where it promotes the production of inflammatory mediators, insulin resistance, skeletal muscle protein consumption, and muscle fiber atrophy, and decreases skeletal muscle mass and content, which reflects the etiology and pathogenesis of sarcopenia. Accordingly, pentadecanoic acid may represent a predictive biomarker for DS.

5’-MTA levels were also elevated in the DS group compared to the DM group. MTA is a naturally occurring hydrophobic thioadenine nucleoside (28), which is an intermediate metabolite or by-product of the methionine (MET) cycle and polyamine synthetase, both of which are intricately associated with key cellular pathways, including gene expression regulation, proliferation, differentiation and apoptosis (29). Although MET is a substrate for homocysteine, elevated levels of MET can cause hyperhomocysteinemia (30), which has been shown to reduce skeletal muscle cell viability and produce an energy imbalance (31–33). Moreover, elevated plasma homocysteine can lead to different systemic or local neurological diseases and promote inflammation (34). Hence, hyperhomocysteinemia can cause skeletal muscle injury and dysfunction, as well as reduce skeletal muscle development and growth (35). Therefore, an increase in MET increases sarcopenia to a certain extent, which is consistent with our experimental results.

In our study, ADMA concentrations were higher in DS patients than in DM controls. ADMA, a naturally occurring chemical found in the plasma, is a metabolic by-product of the protein modification process in the human cytoplasm and is closely related to L-arginine (L-Arg) (36). L-Arg is a conditionally essential amino acid and precursor of nitric oxide (NO) (37). As a key endogenous regulator of vascular tone and endothelial function, NO plays an important role in the regulation of sympathetic vasoconstriction in the microcirculation of exercise muscles (38). ADMA has also been shown to inhibit NO production by L-arginine *via* inhibition of NO synthase production (39), thus leading to an imbalance in vascular contraction and diastolic function of skeletal muscle, blood hypoperfusion, tissue ischemia, and hypoxia. Consequently, skeletal muscle protein formation is negatively impacted, cell and skeletal muscle cells begin to atrophy, and the occurrence and development of sarcopenia is exacerbated. In addition, ADMA can promote the development of chronic inflammation, which can cause damage to skeletal muscle cells. Meanwhile, inflammatory factors also impact ADMA homeostasis (40). Regarding DS, in particular, the enhanced inflammatory response and increased activity of inflammatory factors lead to a decrease in the Arg/ADMA ratio (41), which increases ADMA synthesis and exacerbates the development of diabetes-related muscle mass, strength, and functional decline.

We also found that glutamine concentrations were higher in DS patients than in DM controls. Glutamine is the most abundant amino acid in the blood. As a non-essential amino acid, it is mainly catalyzed by glutamine synthase to synthesize ammonia (NH3) and glutamate in skeletal muscle and is involved in myriad metabolic pathways, including signal transduction, nitrogen transport, energy metabolism, etc. (42, 43). Under stimulation by sarcopenia and hyperglycemia, the body enters a state of long-term, low-level inflammation, causing the abundance of leukocytes, such as lymphocytes, to increase. Unlike skeletal muscle, white blood cells do not have glutamine synthase and cannot synthesize glutamine. In addition, macrophages, lymphocytes, and other inflammatory cells will consume glutamine as a substrate. Therefore, to meet the demand for glutamine anabolism under inflammatory conditions, skeletal muscle cells increase their glutamine production, which becomes released into the blood, causing an increase in plasma glutamine concentration. Indeed, Ilaiwy et al. observed that *in vitro* muscle cell atrophy is related to an increase in glutamine concentration in culture media (44). Moreover, considering that the DS group was older than the DM group and aging is defined as a disease of anabolic resistance to nutrients (such as amino acids), these patients may have exhibited impaired protein synthesis of glutamine, resulting in reduced muscle protein synthesis and development of sarcopenia. Based on these collective study results, we postulate that glutamine concentration is elevated in the DS group and may serve as an effective metabolic marker of DS.

In addition, we found that IX levels were lower in DS patients than in the DM group. IX is a prenyl flavonoid that inhibits the production of secondary bile acids. Secondary bile acids are cytotoxic DNA-damaging metabolites converted from primary bile acids by 7-α-dehydroxylase (45) that interfere with proper metabolism of sugars and lipids, causing lipids to accumulate in muscle tissues, induction of oxidative stress and the inflammatory response, mitochondrial dysfunction, insulin resistance, and damage to skeletal muscle cells, thereby increasing the risk of developing DS (46). Moreover, xanthohumol, a precursor of IX, has been shown to have anti-obesity effects in animal studies (47). Therefore, IX might improve glucose and lipid metabolism to a certain extent, thereby improving lipid accumulation in diabetes and reducing the possibility of DS.

We also found that 3-methylxanthine was highly expressed in the DS group. 3-Methylxanthine is nephrotoxic in that it impacts kidney function, causing kidney damage and increasing the risk of end-stage renal disease (48). Decreased renal function, as an independent risk factor for sarcopenia, suggests that 3-methylxanthine appears to be a predictive metabolite for diabetic myopathy (49).

The level of L-(+)-arginine was also significantly higher in the DS group than in the DM control group. L-(+)-arginine is an inducer of mTOR, which induces muscle protein synthesis primarily through the mTOR pathway (50). However, studies have shown that when sarcopenia occurs, the L-(+)-arginine concentration in muscles begins to decrease (51). Hence, we postulate that our findings may have resulted from protein decomposition in plasma and a subsequent compensatory increase in L-(+)-arginine concentration, reflecting metabolic changes secondary to sarcopenia.

We also observed a significant increase in D-gluconic acid levels within the DS group. D-Gluconic acid is involved in the antioxidative stress response, thereby upregulating glycolysis and downregulating the TCA cycle, leading to mitochondrial dysfunction and muscle ATP deficiency (52, 53). However, due to the lack of further evidence related to sarcopenia, more studies are needed to verify its applicability as a metabolic marker for DS.

We further identified metabolic pathways associated with DS using KEGG enrichment analysis. Among the enriched pathways were ABC transporter superfamily, mTOR signaling pathway, Chagas disease, amoebiasis, amyotrophic lateral sclerosis, pyrimidine metabolism, D-arginine and D-ornithine metabolism, as well as seven other metabolic pathways. Of these pathways, ABC transporters and the mTOR signaling pathway are the most influential and may represent potential target pathways for DS. ABC transporters, one of the largest families of membrane proteins in most organisms, are involved in myriad key physiological processes and serve as pathogenic factors in many diseases. In fact, mutations in ABC-related genes cause neurological disorders and defects in cholesterol and bile transport (54). Cholesterol transport defects affect lipid metabolism and lead to fat deposition in skeletal muscle, thus affecting the activity of skeletal muscle cells and causing skeletal muscle atrophy. Meanwhile, neurological diseases directly affect the activity of innervated muscles, thus impacting communication between muscles and nerves, as well as the types of muscle fibers and expression of myosin (55). This causes muscle weakness and atrophy, which increases the risk of developing sarcopenia. The mTOR signaling pathway plays a key regulatory role in mitochondrial autophagy in skeletal muscles. Mitochondrial quality in skeletal muscle cells is closely associated with sarcopenia; reduced quality deteriorates the muscle microenvironment, leading to the progression of sarcopenia in naturally or rapidly aging mice (56). In addition, L-(+)-arginine also has an important role in the mTOR signaling pathway. Hence, we purport that ABC transporters and the mTOR signaling pathway may contribute to DS development and, therefore, may represent target pathways for the prevention and treatment of DS.

Certain limitations were noted in this study. First, the relatively small sample size limited the statistical ability to definitively identify metabolites associated with DS. Second, due to the cross-sectional design, we were unable to predict DS prognosis or determine the causal relationship between serum differential metabolites and DS. Further studies are warranted to elucidate the clinical correlation between these metabolites and DS, as well as the related mechanisms. Nevertheless, the advantage of our study lies in the application of metabolomics analysis to identify potential metabolites related to the development and progression of DS. These findings may inform the development of effective preventative and therapeutic modalities for DS.

## 5 Conclusion

In conclusion, this study explored potential biomarkers of DS using a metabolomic strategy. Pentadecanoic acid, 5’-MTA, ADMA, and glutamine were identified as potential biomarkers of DS, while ABC transporters and the mTOR signaling pathway likely make significant contributions toward DS development. The potential relationships between differential metabolites and DS provide novel insights into the mechanisms underlying DS and potential strategies for future treatments.

## Data availability statement

The raw data supporting the conclusions of this article will be made available by the authors, without undue reservation.

## Ethics statement

The studies involving human participants were reviewed and approved by the Medical Ethics Committee of the Qilu Hospital of Shandong University. The patients/participants provided their written informed consent to participate in this study. Written informed consent was obtained from the individual(s) for the publication of any potentially identifiable images or data included in this article.

## Author contributions

YT Conceptualization, Methodology, Resource, Investigation, Formal analysis, Data curation, Visualization, Writing - Original draft and Writing - Review & Editing. XL Data curation and Formal analysis. YY Data curation and Formal analysis. BL Conceptualization, Validation and Writing - Review & Editing. YF Data curation and Formal analysis. WZ Data curation. CF Data curation. XY Data curation. ZH Formal analysis and Funding acquisition. MC Supervision, Funding acquisition, Resources and Project administration. All authors contributed to the article and approved the submitted version.
